# Climate and Suicide

**Published:** 1888-08-25

**Authors:** 


### Climate and Suicide.

Frenchmen who visit England for the first time express astonishment that we who live here always do not all commit suicide in a body. There is no doubt that if an Englishman be of a railing disposition, he will find abundance of occupation for his talent in the vagaries of the climate between January and December. Not only is it cold when it should be hot, and foggy when it should be clear, but the rain pours down in the most irrational and inconsequent manner at all seasonable and unseasonable times. Poor Mrs. Smith has been waiting all the present summer, for example, to give her first garden party. Week after week the rain and the cold have deliberately conspired to drive her wild. At length come two or three days of brilliant sunshine, and her excitement reaches its maximum. All her invitations are despatched by a single post, and everything is going to be a success in the end. The sunshine continues, the day approaches, the refreshments are ordered. At last the morning breaks: the wind has veered round in the night; the sky is overcast, but still there is no rain. How she watches those leaden clouds as the hours creep on! Will they never break? If they would only do one thing or another—either pour down the torrents they are holding back, or fly away with the gusty wind to some district where no garden party is in anticipation! But, no! Mrs. Smith is certain the rain will obstinately keep off all day until every guest has arrived, and then come down in a perfect deluge. That is exactly what happens. No sooner has the vicar's wife brought up the rear with her three charming daughters, and the doctor finished his apology for being so late, then down it comes like a vicious and determined Noah's flood.

It is provoking—most provoking. It may even be said to be disgusting. And the worst of it is that there is no help for it whatever. It is of no use to scold the barometer or the Astronomer Royal, or even the refreshment contractor. There is the miserable rain, and there it is certain to continue; and there is positively nothing else for it but to cram all the guests, wet, disappointed, and cross into the drawing-room. What materfamilias born and bred in these islands is equal to the entertainment of a garden party in a drawing-room? As Carlyle says: "Imagination will not forward with the picture."

Examples similar to poor Mrs. Smith's could be multiplied by the score. The man of business hurrying into the City in his early morning train has seen at the various stations, during the summer, scores of Sunday-school parties bent on country excursions, all soaked and bedraggled before they left the smoke and din of the town. How he has pitied them! Though it must be admitted they have seldom seemed to feel much pity for themselves. Their youthful energy and cheery spirits have triumphed over both cold and rain. The patriotic cricketer, bent on making large scores, has been ignominiously bowled in the first "over." The cyclist, with his last new suit and his machine in perfect order, has waited in vain for his long-anticipated tour. The farmer has been compelled to stand idly by whilst his hay has rotted in the fields, and his finest wheat crops have been levelled to the ground. Business and pleasure have had to be suspended; young and old have been alike disappointed and disgusted.

Strange to say, there have been very few suicides, and those which have occurred seem to have had little to do with the weather. We are challenged by foreigners that we ought to commit suicide in such a climate as ours, even in its normal condition. What then ought we to have done during the last four or five months? According to the theorists, there should not have been a rope, nor a razor, nor a drop of prussic acid, nor a single live person left above the age of fifteen. If ever an experiment on climate and suicide was performed on a large scale it has been performed during the recent spring and summer. Never since the year 1809 has there been so uniformly cold, wet, foggy, and miserable a season. Yet, where are the suicides? The fact of the matter seems to be that climate, in itself, has nothing whatever to do with suicide.

The present is a good opportunity for calling attention to this comprehensive experiment. So far as we have been able to ascertain by a careful study of the newspapers there has been no increase in the number of suicides whatever. There will, however, be reliable data forthcoming at no distant date, when the Registrar-General publishes his annual report; and those who are curious in such matters, as we all should be, may confirm or refute the opinions here set forth.

But though we may acquit our skies and clouds and winds of manslaughter, we cannot deny that they are responsible for a good many ailments and troubles of a very annoying and sometimes serious character. The east wind on a clear March day may be bracing and even pleasant; but when it turns its biting attention to us in Augnst, and brings showers in its cold and shivering train, it is another thing. In March we are armed with Jaeger's flannels and woollen overcoats; in August we are clothed in delusive and defenceless alpaca. What wonder is it that livers go wrong and tempers become acid? what wonder that catarrhs invade the nostrils and rheumatism the joints?

In such a season as this a dignified conservatism is at a discount. We cannot stick to the old ways. We must change with the times. Even the most rigid stickler for white hats and dust-coats will consent to put on a woollen overcoat as he takes his fifty miles' journey to Brighton at seven o'clock in such bitter evenings as we have had lately. And this is really, like the postscript in a lady's letter, the immediately practical point of the whole paper. In a changeable climate like ours, we should be prepared to jump from summer to winter in our attire at half-an-hour's notice. This is absolutely essential, especially for delicate persons and children. Fashion and custom must be voted nuisances, and everything must give way to health. Those who will intelligently adapt themselves to the necessities of the moment may find themselves at the end of the summer, not only as well, but even better than usual. Those who refuse to do so will pay the penalty in colds, coughs, rheumatism, disordered liver, and a host of ills which, if not fatal, are both injurious and costly, and the very worst preparation for the coming season of winter.

We may yet, perhaps, have a few weeks of summer, though the prospect is of the dreariest. It will be best to prepare for a long autumn of short, cloudy days, and long chilly evenings and nights. Under flannels should, at any rate, be resumed. Probably in many cases they have never been left off. There is some compensation in the hope that if we get well accustomed to the cold now, some of us will avoid the colds in the head and chest which are so frequently for us the first harbingers of winter. There are very few evils which have not their compensations; and if any who have been compelled to wear autumn or winter clothing all through the summer find themselves better seasoned than usual when the frosts and fogs and rains of November have them in their grasp, they may not perhaps be sorry that some garden parties were failures, and that tennis tournaments were things impossible.

